# Electron Paramagnetic Resonance Highlights That the Oxygen Effect Contributes to the Radiosensitizing Effect of Paclitaxel

**DOI:** 10.1371/journal.pone.0040772

**Published:** 2012-07-12

**Authors:** Fabienne Danhier, Pierre Danhier, Nicolas Magotteaux, Géraldine De Preter, Bernard Ucakar, Oussama Karroum, Bénédicte Jordan, Bernard Gallez, Véronique Préat

**Affiliations:** 1 Pharmaceutics and Drug Delivery, Louvain Drug Research Institute, Université Catholique de Louvain, Brussels, Belgium; 2 Laboratory of Biomedical Magnetic Resonance, Louvain Drug Research Institute, Université Catholique de Louvain, Brussels, Belgium; Pennington Biomedical Research Center, United States of America

## Abstract

**Background:**

Paclitaxel (PTX) is a potent anti-cancer chemotherapeutic agent and is widely used in the treatments of solid tumors, particularly of the breast and ovaries. An effective and safe micellar formulation of PTX was used to administer higher doses of PTX than Taxol® (the current commercialized drug). We hypothesize that PTX-loaded micelles (M-PTX) may enhance tumor radiosensitivity by increasing the tumor oxygenation (pO_2_). Our goals were (i) to evaluate the contribution of the “oxygen effect” to the radiosensitizing effect of PTX; (ii) to demonstrate the therapeutic relevance of the combination of M-PTX and irradiation and (iii) to investigate the underlying mechanisms of the observed oxygen effect.

**Methodology and Principal Findings:**

We used (PEG-p-(CL-*co*-TMC)) polymeric micelles to solubilize PTX. pO_2_ was measured on TLT tumor-bearing mice treated with M-PTX (80 mg/kg) using electron paramagnetic resonance (EPR) oximetry. The regrowth delay following 10 Gy irradiation 24 h after M-PTX treatment was measured. The tumor perfusion was assessed by the patent blue staining. The oxygen consumption rate and the apoptosis were evaluated by EPR oximetry and the TUNEL assay, respectively. EPR oximetry experiments showed that M-PTX dramatically increases the pO_2_ 24 h post treatment. Regrowth delay assays demonstrated a synergy between M-PTX and irradiation. M-PTX increased the tumor blood flow while cells treated with M-PTX consumed less oxygen and presented more apoptosis.

**Conclusions:**

M-PTX improved the tumor oxygenation which leads to synergy between this treatment and irradiation. This increased pO_2_ can be explained both by an increased blood flow and an inhibition of O_2_ consumption.

## Introduction

Paclitaxel (PTX) is a potent anti-cancer chemotherapeutic agent and is widely used in the treatments of solid tumors, particularly of the breast and ovaries [1]. PTX promotes the polymerization of tubulin causing the death of the cell by disrupting the normal tubule dynamics required for the mitotic cell division [2].

Radiotherapy is most effective in controlling local-regional disease whereas chemotherapy can attack distant metastases. Furthermore, many chemotherapeutic agents are able to increase the sensitivity of tumors to radiation [3]. Besides its anti-tumor activity, PTX has been reported as a radiosensitizing agent both *in vitro* and *in vivo* [3], [4]. This was firstly based on the principle that the PTX blocks cells in the G_2_/M phase which is the most radiosensitive of all cell cycle phases. Moreover, Milas *et al*. suggested that the PTX-induced enhancement of tumor radioresponse was also mediated by reoxygenation of hypoxic tumor cells [3], [5]. Oxygen deficiency in tumors reduces the efficacy of cancer treatments such as chemotherapy or radiotherapy. Hypoxia has been described as an important factor for development of tumor aggressiveness and there is an overlap between this phenomenon and the poor outcome of hypoxic tumors to radiotherapy [6]. Using radiotherapy, the presence of molecular oxygen increases DNA damage through the formation of oxygen-derived free radicals, which occurs primarily after the interaction of radiation with intracellular water. Because of this so-called “oxygen effect”, the radiation dose required to achieve the same biologic effect is about three times higher in the absence of oxygen than in the presence of normal levels of oxygen [7]. The level of tumor oxygenation depends on the balance between oxygen supply, as result of inadequate tumor perfusion, and consumption (mainly via mitochondrial respiration) [8].

PTX is currently commercialized (Taxol®) in mixture of Cremophor® EL and ethanol (50∶50, v/v). This excipient is responsible for a lot of serious side effects such as hypersensitivity reaction and neuropathy [1]. Recently, novel polymeric micelles have been developed as safe and effective delivery system for PTX [9]. Removing Cremophor® EL of the formulation, these polymeric micelles have allowed the enhancement of the Maximum Tolerated Dose (MTD) of Taxol® from 13.5 mg/kg to 80 mg/kg, after intraperitoneal injection. Polymeric micelles are known to extravasate from blood vessels to tumors through fenestrations presented in the tumor endothelium and to be retained into the tumor tissue, by the well-known Enhanced Permeation and Retention effect [10], [11]. Because of the increased resident time in the tumor resulting from the Enhanced Permeation and Retention effect and because of the solubilization of PTX in polymeric micelles (avoiding the Cremophor® EL-associated toxicity of Taxol®), we hypothesized that the use of polymeric micelles could be considered as a innovative tool to study the radiosensitivity of PTX.

The aims of this work were (i) to evaluate if the “oxygen effect” using electron paramagnetic resonance (EPR) oximetry could be one of the mechanisms involved in the radiosensitizing effect of PTX-loaded micelles; (ii) to demonstrate the therapeutic pre-clinical relevance of the combination of PTX-loaded micelles and irradiation and (iii) to investigate the underlying mechanisms of the observed oxygen effect. All experiments were performed on TLT tumor-bearing mice. This tumor model was chosen because it is a fast growing model which presents intrinsic hypoxia-mediated resistance which is described as an important cause of unsuccessfulness of radiotherapy [6], [7].

## Methods

### 1. Preparation and Characterization of Micelles Loaded with PTX

PTX-loaded micelles were prepared as previously described [12], [9]. The amount of solubilised PTX in micelles was determined by HPLC. The particle size and zeta potential were determined, respectively, by photon correlation spectroscopy (PCS) and laser Doppler velocimetry [9].

### 2. Tumor Model

Syngeneic TLT tumors [13], [14] were injected intramuscularly in the gastrocnemius muscle in the rear leg of 8 week old male NMRI mice. Before injection, animals were anesthetized with a Ketamine/Xylazine dose mixing (62.5 and 6.25 mg/kg, respectively). All experiments were performed in compliance with guidelines set by national regulations and were approved by the Ethical Committee for animal care of the Université Catholique de Louvain (Permit number: UCL/MD/2008/025). All tumor implantation were performed under anesthesia and all efforts were made to minimize suffering.

### 3. pO_2_ Measurements by Electronic Paramagnetic Resonance (EPR) Oximetry

Tumor oxygen measurements were performed on TLT tumors bearing mice, using Electron Paramagnetic Resonance (EPR) oximetry. Treatments began when tumors reached 8.0±0.5 mm in diameter. PTX-loaded micelles were injected intravenously (13.5 mg/kg) or intraperitonealy (80 mg/kg); 2, 4, 6, 20 and 24 h before pO_2_ measurements. These times were chosen based on the literature [3], [5]. Control mice were not treated. EPR spectra were acquired by an EPR spectrometer (Magnettech) with a low-frequency microwave bridge operating at 1.2 GHz and extended loop resonator. For oxygen detection *in vivo*, charcoal (Charcoal wood powder, CX0670-1, EM Science) was used as suitable sensor interacting with oxygen. Mice were injected 24 h before EPR analysis in the center of the tumor using a suspension of charcoal (100 mg/ml, 1–25 µm particle size). The tumor was placed in the center of the extended loop resonator which had a sensitive volume extending 10 mm^3^ into the tumor mass, using a protocol previously described [14], [15].

### 4. Irradiation and Tumor Regrowth Delay Assay

The effect of PTX-loaded micelles combined with X-rays applied 24 h after chemotherapy on TLT growth was assessed by daily measurements of the diameter of the tumors with an electronic calliper. The tumor-bearing leg was locally irradiated with a single dose of 10 Gy of 250-kV X-rays (RT 250, Philips medical Systems). When TLT tumors reached 7.0±0.5 mm in diameter, the mice were randomly assigned to a treatment group: group 1, PBS injection; group 2, PTX-loaded micelles (PTX dose of 80 mg/kg); Group 3, irradiation with 10 Gy of X-rays; Group 4, PTX-loaded micelles (PTX dose of 80 mg/kg) + irradiation with 10 Gy of X-rays 24 h post chemotherapy; Group 5, PTX-loaded micelles + irradiation with 10 Gy of X-rays 24 h post chemotherapy (mice leg ligatured).

### 5. Underlying Mechanisms of the Improvement of Tumor Oxygenation

#### 5.1 Evaluation of tumor perfusion by Patent blue staining

The Patent blue (Sigma-Aldrich) was used to estimate the tumor perfusion (adapted from [16], [17]) of mice pre-treated with PTX-loaded micelles (80 mg/kg) 24 h before. We injected 50 µl of patent blue stain (1.25%) into the tail vein of mice. After 1.0 min, mice were sacrificed and the tumor was carefully excised and cut into two halves. Pictures of each tumor cross section were taken with a digital camera. The comparison between the stained and the unstained area was performed using an in-house program running on Interactive Data Language (Research Systems Inc. Boulder). For each tumor, the stained area was defined on two pictures and the percentage of stained area of the whole cross section was calculated.

#### 5.2 *Ex vivo* oxygen consumption rate evaluation by EPR oximetry

The oxygen consumption was assessed using an EPR method [18]. The spectra were recorded on a Bruker EMX EPR spectrometer operating at 9 GHz. TLT tumor-bearing mice were treated with PTX-loaded micelles (80 mg/kg) (untreated mice were used as control) and were sacrificed 24 h post-treatment. Tumors were excised, trysinized for 30 min and the cell viability was determined. Cells (1.5×10^7^/ml) were suspended in 20% dextran in complete medium. A neutral nitroxide, ^15^N 4-oxo-2,2,6,6-tetramethylpiperidine-d_16_-^15^N-1-oxyl at 0.2 mM (CDN Isotopes), was added to 100 µl of tumor cells that were then drawn into glass capillary tubes. The probe was calibrated at varying oxygen levels between 100% nitrogen and air so that the linewidth measurements could be related to oxygen at any value. The sealed tubes were placed into quartz EPR tubes and the samples maintained at 37°C. Oxygen consumption rates were calculated by measuring the pO_2_ in the closed tubes as a function of time and subsequently finding the slope of the resulting linear plot [19].

#### 5.3 Apoptosis in TLT tumor treated by PTX-loaded micelles

Apoptosis in TLT-tumors after PTX-loaded micelles was evaluated by the TUNEL assay. 24 hours after injection of PTX-loaded micelles (80 mg/kg) (untreated mice were used as control), TLT-tumor bearing mice were sacrificed. Tumors were removed and covered with cryo-embedding compound OCT (Tissue-Tek). 5 µm-sections were mounted onto charged superfrost-plus glass slides and stored at −80°C until staining. Slides were then fixed with a paraformaldehyde 4% solution and permeabilized with Triton X-100 0.1%. The fluorescent TUNEL assay was then conducted by following instructions of In Situ Cell Death Detection kit (Roche). Nuclear apoptosis is accompanied by the segmentation of nucleus into dense nuclear parts further distributed into apoptotic bodies. These DNA breaks can be visualized by red-labeled TdT staining. Nuclei were stained using Hoechst 33342 (Sigma-Aldrich), mounted with Vectashield (Vector Laboratories) and examined under a fluorescent microscope with 350 nm (blue) and 515–560 nm (red) excitation filters.

### 6. Statistics

**Figure 1:**
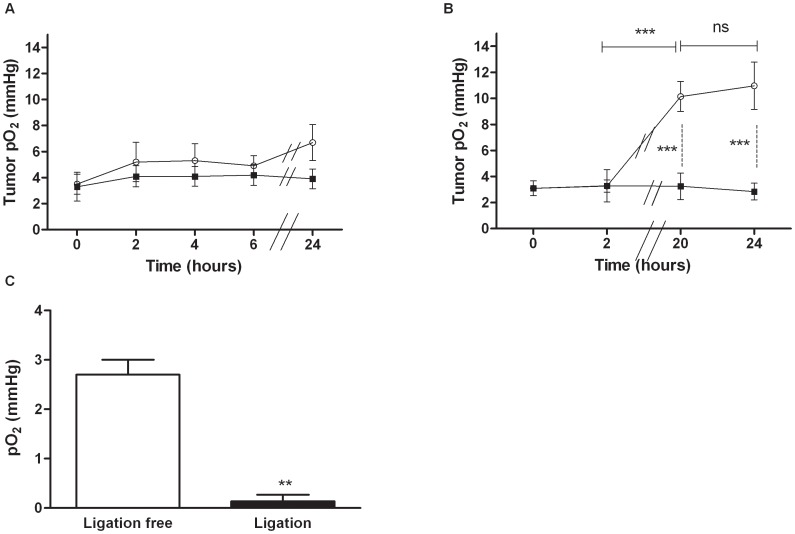
Effect of the administration of PTX-loaded micelles on tumor pO_2_. Mean tumor pO_2_ (± SD) monitored by EPR oximetry. A. Mice treated with PTX-loaded micelles (13.5 mg/kg) (○); Non treated mice (▪) (*n* = 4 for 0, 2, 4 and 6 h; *n* = 3 for 24 h) B. Mice treated with PTX-loaded micelles (80 mg/kg) (○); Non treated mice (▪) (*n* = 7 except for control mice at 24 h: *n* = 4). C. Effect of the ligation of leg of mice on tumor pO_2_ (*n* = 3). **p<0.01, ***p<0.001.

All results are expressed as mean ± standard error of the mean (SEM) except for Fig. 1 where results are expressed as mean ± standard deviation (SD). Two-way ANOVA and Bonferroni post test, t-test, linear regression and Kaplan-Meier survival rate were performed using the software GraphPad Prism 5 for Windows to demonstrate statistical differences (*p*<0.05).

## Results

### 1. Preparation and Characterization of Micelles Loaded with PTX

A solution of 10% of PEG-p(CL-*co*-TMC) allowed injections of PTX at a dose of 13.5 mg/kg, intravenously and 80 mg/kg, intraperitonealy. PTX-loaded micelles had a size and a zeta potential of 24.1±0.5 nm and −2.8±0.6 mV, respectively [9].

### 2. Enhancement of Tumor pO_2_ Induced by PTX-loaded Micelles

EPR oximetry relies on the oxygen-dependent broadening of the EPR line width of a paramagnetic oxygen sensor implanted in the tumor. This technique allows repeated measurements from the same site for days and weeks [7]. Intravenous injection of PTX-loaded micelles (13.5 mg/kg) did not change the tumor pO_2_ when compared to non treated mice (*p*>0.05). Nevertheless, there was a slight enhancement (not significant) for mice treated 24 h before the pO_2_ measurement (Fig. 1A). Based on the literature [20] and this result, we hypothesized that an increase of pO_2_ could be observed at 24 h with a higher dose of PTX. Intraperitoneal injection of PTX-loaded micelles (80 mg/kg) dramatically modified the tumor pO_2_ in TLT tumors as shown in Fig. 1B. The tumor pO_2_ before the treatment was 3.1±1.5 mm Hg. A significant increase (*p*<0.001) in pO_2_ was observed from 20 h (10.15±3.07 mm Hg) and remained elevated until at least 24 h (10.97±4.8 mm Hg).

### 3. Synergy between PTX-loaded Micelles and Irradiation

**Figure 2:**
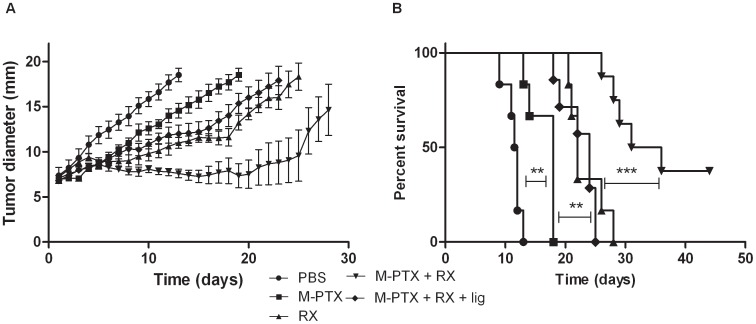
Anti-tumor effect on TLT-tumor-bearing mice treated with PTX-loaded micelles in combination with irradiation. (A) Anti-tumor effect. PTX is administered at a dose of 80 mg/kg. Six groups of mice were treated: Group 1: PBS injection (*n* = 6); Group 2: PTX-loaded micelles (*n* = 6); Group 3: Irradiation with 10 Gy of X-rays (*n* = 6); Group 4: PTX-loaded micelles 24 h before irradiation with 10 Gy of X-rays (*n* = 8); Group 5: PTX-loaded micelles 24 h before irradiation with 10 Gy of X-rays. The irradiated legs of mice were clamped (*n* = 7); Each point represents the mean of tumor size ± SEM. (B) Survival rates of tumor-bearing mice. (C) Individual evolution of the TLT growth as a function of time. ** *p*<0.001; ***p<0.0001.

Based on the observed increased tumor oxygenation assessed by EPR oximetry, we checked whether PTX-loaded micelles may enhance tumor radioresponse. Hence, the in vivo anti-tumor efficacy of the combination of PTX-loaded micelles and irradiation was performed (Fig. 2). PTX-loaded micelles were administered 24 h before irradiation, based on EPR oximetry results. All treatments were significantly different as compared to the PBS control group (*p*<0.001). Previously, it was demonstrated that unloaded micelles did not present any sign of cytotoxicity both *in vitro* and *in vivo* [11]. The time to reach 18 mm was 14 days for mice treated with PTX-loaded micelles and 21 days for irradiated mice (*p*<0.001). The combination of these two treatments allowed a significant enhancement of the time to reach 18 mm until 28 days when compared with single treatment (PTX-loaded micelles or irradiation, *p*<0.001 and p<0.01, respectively). Mice were also treated with the combination of treatments with a transient ligation, using a rubber band, (just before and during the irradiation) of their leg to abolish the oxygen effect caused by PTX-loaded micelles. Interestingly, with the ligation, the time to reach 18 mm was decreased to 19 days which is similar to mice treated only with irradiation (*p*>0.05). The effect of ligation was also confirmed by pO_2_ measurements (Fig. 1C). On the other hand, no significant difference was observed when TLT-bearing mice were irradiated and when mice were irradiated with the ligation of their leg (*p*>0.05). Indeed, TLT tumors are hypoxic (pO_2_<5 mm Hg [6]. Consequently, the ligation of the legs did not significantly decrease the oxygenation of hypoxic tumors. Together these results indicate that the ligation of PTX-treated tumors induced anoxia and counteracted the radiosensitizing effect of PTX and that the oxygen effect may contribute to the radiosensitizing properties of PTX (Fig. 2A).

The survival rate was significantly higher for mice treated with the combination of PTX-loaded micelles and irradiation as compared to other groups (*p*<0.001). After 45 days, for the half of mice, it was not anymore possible to detect any tumor (Fig. 2B).

### 4. Underlying Mechanisms of the Improvement of Tumor Oxygenation

To understand which mechanisms are responsible of the observed improvement of tumor oxygenation 24 h after PTX-loaded micelles treatment, additional experiments were performed: (i) the patent blue staining was used to evaluate the tumor perfusion, (ii) the oxygen consumption rate of tumor was assessed ex vivo by EPR oximetry and (iii) the apoptosis induced by PTX-loaded micelles was examined by fluorescent microscopy, using the TUNEL assay.

#### 4.1 Tumor perfusion

**Figure 3:**
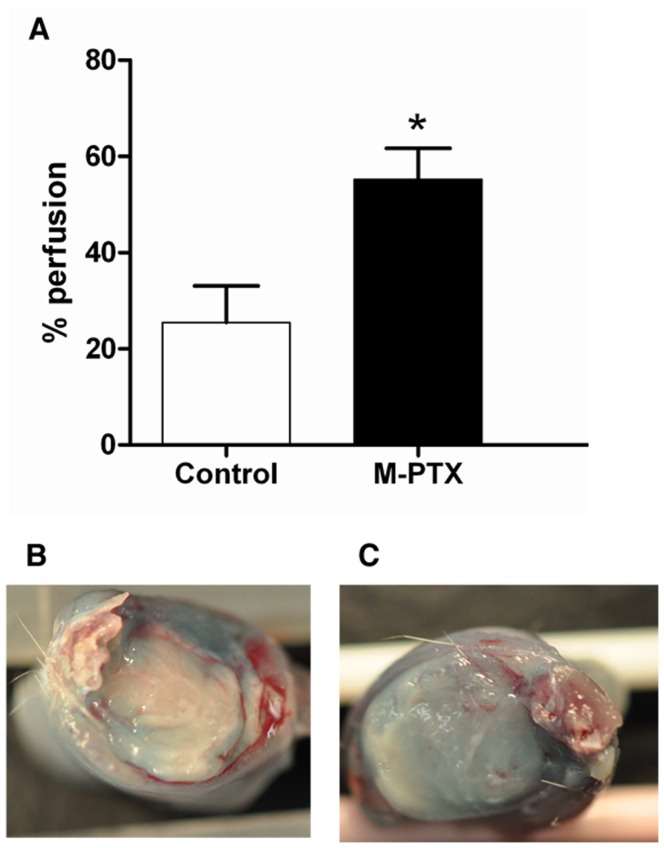
Effect of PTX-loaded micelles on tumor perfusion 24 h post treatment by Patent blue staining. (A) Each bar represents the mean value of tumor percentage of colored area ± SEM for control group and PTX-loaded micelles group. * *p*<0.05. (B) Control. (C) PTX-loaded micelles.

The tumor perfusion was assessed by observing colored area after injection of Patent blue. This method, previously validated in comparison with DCE-MRI, provides an approximate of relative blood flow [21]. Tumors pre-treated 24 h before with PTX-loaded micelles showed a higher stained area, expressed in percentage of perfusion, (*n* = 8; 55.2±6.5%) than non-treated tumors (*n* = 7; 25.4±7.6%) (*p*<0.05), indicating that PTX-loaded micelles increased the tumor blood flow (Fig. 3).

#### 4.2 Tumor oxygen consumption

**Figure 4:**
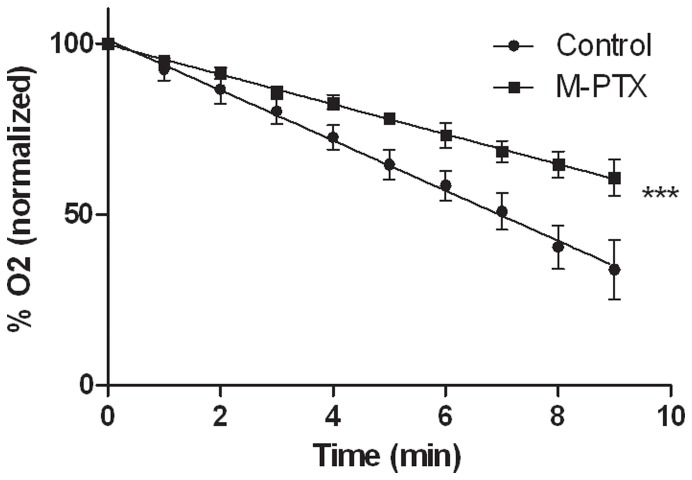
Effect of PTX-loaded micelles 24 h pre-treatment (80 mg/kg) on TLT tumor oxygen consumption rate. Oxygen rate measured as function of time (mean ± SEM). Slope for treated mice (*n* = 3) significantly different than for control mice (*n* = 3). *** *p*<0.001.

We measured by EPR the oxygen consumption rate of tumor cells extracted from TLT tumors of mice treated 24 h before with PTX-loaded micelles (80 mg/kg), compared to the rate of non-treated mice as control group. The mean slopes were found to be significantly different (–4.37±0.05 and –7.34±0.14 for control and treated groups, respectively) (*p*<0.001) (Fig. 4). This means that tumor cells pretreated with PTX-loaded micelles consumed oxygen 1.7 times slower than non-treated tumor cells.

#### 4.3 Apoptosis in TLT tumor treated by PTX-loaded micelles

**Figure 5:**
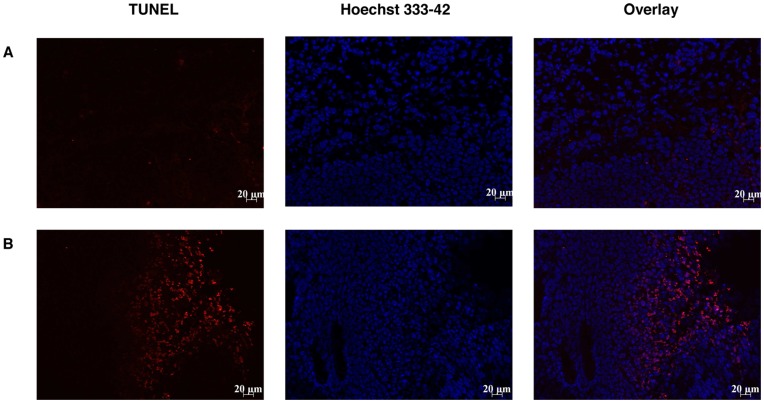
Effect of PTX-loaded micelles 24 h pre-treatment on TLT tumor apoptosis assessed by TUNEL assay. (A) Control mice (*n* = 3). (B) Mice treated 24 h before analysis with PTX-loaded micelles (80 mg/kg). DAPI staining was used to label nuclei (blue) and TUNEL staining was used to label apoptotic cells (red).

Using the TUNEL method to assess apoptosis, visualization of total apoptotic cells (TUNEL positive cells labeled in red) indicated that higher apoptosis was obtained when mice were treated with PTX-loaded micelles compared to untreated mice (Fig. 5).

## Discussion

PTX was previously described as a radiosensitizer agent. Because PTX-treated cells are arrested in the G_2_/M phase and because this phase of the cell cycle is known to be the more radiosensitive, a combination of PTX with ionizing radiation has been proposed [3]. Moreover, tumor reoxygenation has been suggested to be another important mechanism by which PTX potentiates the radioresponse of tumors [5]. Elsewhere, it has been previously suggested that these two mechanisms are strongly linked: the tumor reoxygenation resulting from the G_2_/M block induced by PTX [5].

PEG-p-(CL-*co*-TMC) polymeric micelles loaded with micelles were recently described as safe and effective PTX delivery system, which drastically enhance the maximum tolerated dose (MTD) of Taxol® [9]. Two PTX-loaded micellar systems are currently in clinical trials: Genexol-PM (phase IV for breast cancer) and NK105 (phase II for stomach cancer). For example, Genexol allows a 3-fold enhancement of the MTD while our system allows a 6-fold enhancement of the MTD. Moreover, compared to the most of polymeric micelles described in the literature, the major advantage of our PEG-p-(CL-*co*-TMC) micelles is that they are self-assembling upon contact with water and that not organic solvent neither dialysis or freeze-dried or evaporation steps is required leading this system feasible for scaling-up.

To evaluate if the radiosensitizing effect of PTX-loaded micelles results from an oxygen effect, the evaluation of reoxygenation over the time should be established. Therefore, we assessed EPR oximetry after injection of PTX-loaded micelles (80 mg/kg). After 20 h, a drastic increase in pO_2_ was observed and was maintained at least until 24 h (the critical cut off of pO_2_ is situated at 5 mm Hg). Since cytotoxic effects, after a single PTX dose, decrease after 3 days [9], we can thus suppose that the increased pO_2_ does not last more than 3 days. At the MTD of Taxol® (13.5 mg/kg), no increase in pO_2_ was observed. Polymeric micelles may thus be considered as an innovative tool to administer higher doses of PTX. It is important to note that at this dose of PTX, its was impossible to compare our polymeric micelles formulation with Taxol® because of the death of mice within 2 h treated with Taxol® at the equivalent PTX dose. Moreover, it is important to note that it was previously demonstrated that unloaded micelles did not present any sign of cytotoxicity both *in vitro* and *in vivo* [11]. These Electron Paramagnetic Resonance results allow the determination of the time window of reoxygenation, which was used to design the irradiation experiments. To our knowledge, for the first time, we used the timing sequence of reoxygenation by measurement of pO_2_ to design experiments of PTX treatment combined with irradiation. Using the regrowth delay assay, we found a synergy between PTX and irradiation during the reoxygenation window. The hypothesis that a synergy between PTX and irradiation was mediated with an oxygen effect was clearly confirmed with the regrowth delay assay on TLT tumor-bearing mice treated with PTX-loaded micelles 24 h before irradiation. Tumor clamping by temporary ligation of the irradiated leg induced complete hypoxia at the time of irradiation and prevents enhancement of tumor pO_2_ induced by PTX treatment.The fact that mice treated by the combination of PTX and X-rays, with ligation of their leg, responded similarly to mice treated only with irradiation demonstrates that the radiosensitizing effect of PTX is mainly due to an oxygen effect. We did not expect any difference between irradiation alone group and X-rays + ligature group because the chosen tumor model TLT is already described as highly hypoxic. Using such radioresistant model is thus well adapted to demonstrate oxygen-mediated radiosensitizing properties of PTX [22].

**Figure 6:**
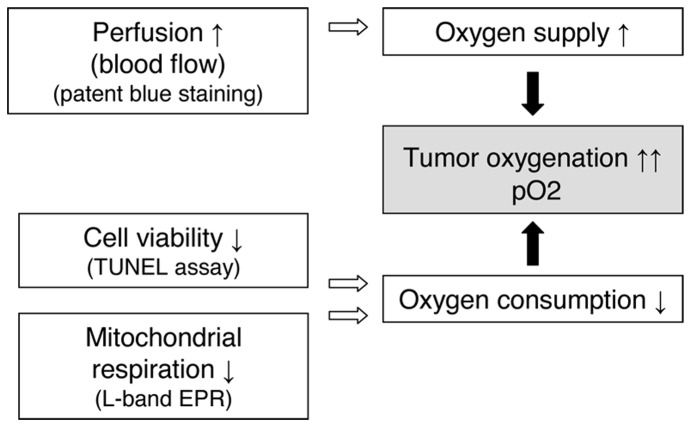
Mechanisms of tumor oxygenation induced by PTX (80 mg/kg). Mechanistically, tumor oxygenation results from an balance between oxygen delivery (perfusion) and local oxygen consumption (which depends on the cellular viability and mitochondrial respiration) (Adapted from [7]).

The enhancement of tumor oxygenation can be explained by either an enhancement in blood supply which is associated with an increased blood flow or/and by a decrease in oxygen consumption (Fig. 6). We demonstrated that PTX-loaded micelles contribute to reoxygenation effect by both of these mechanisms. To our knowledge it is the first study which evaluates the mechanisms underlying the “oxygen effect” for PTX. The radiosensitivity of PTX-incorporating micelles was also studied by Negishi *et al.*, focusing their attention to the ability of PTX to arrest cells in G_2_/M phase [23]. The cytotoxic effect of PTX-loaded micelles was previously demonstrated both *in vitro* and *in vivo* [9]. The decrease in oxygen consumption is the result of two distinct mechanisms. First, as we observed an important apoptosis after treatment (Fig. 5), the total number of tumor cells consuming oxygen is decreased. Moreover, we also observed that the PTX treatment induced a decrease in oxygen respiration by the living tumor cells (Fig. 4). In addition to the effect on oxygen consumption, we observed that the PTX treatment led to an increase in tumor perfusion (Fig. 3). This effect could be linked to the decrease of tumor cells leading to a decrease in interstitial fluid pressure and a consequent increase in tumor perfusion.

The radiosensitizing effect of PTX can thus be explained by at least two mechanisms: the G_2_/M blockade and the tumor reoxygenation. The first one was largely described in the literature [3], [23]; while we highlighted and explained the tumor reoxygenation in the present paper. Moreover, these two mechanisms are likely linked: the G_2_/Mblock leads to apoptosis of cells which is partly responsible from the tumor reoxygenation.

In conclusion, we demonstrated that PEG-p(CL-*co*-TMC) polymeric micelles loaded with PTX radiosensitized TLT tumors by improving the tumor oxygenation. Combining polymeric drug chemotherapy with radiation may have important clinical implications in terms of scheduling and optimization of the therapeutic ratio. We also showed that the increased pO_2_ can be explained both by an increased blood flow and an inhibition of O_2_ consumption.
